# Shaping sustainable perceptions: The role of metaphors in Olympic news discourse

**DOI:** 10.1371/journal.pone.0317380

**Published:** 2025-01-13

**Authors:** Wei Peng

**Affiliations:** School of Foreign Languages, Central South University, Changsha, Hunan, China; Federal University of the ABC: Universidade Federal do ABC, BRAZIL

## Abstract

The dissemination of sustainable development concepts in large international events like the Olympics has garnered great attention. As a major international sports event, the Beijing Winter Olympics served as an important platform for showcasing China’s sustainable development philosophy through its official news coverage. In this context, metaphor, as a powerful cognitive tool, plays a crucial role in shaping public perception and facilitating the dissemination of values by mapping concrete source domains onto abstract target domains. This paper constructs a critical metaphor analysis framework for sustainable development, analyzing the mechanisms by which metaphors map the concepts of social, economic, and ecological sustainability, and their multifaceted roles in conveying policy proposals, ideologies, cultural values, and social group behaviors. The findings indicate that metaphors effectively facilitate public understanding of sustainability by concretizing abstract concepts. In the social dimension, metaphors emphasize fairness, cultural diversity, and social solidarity; in the economic dimension, they highlight resource recycling, technological innovation, and industrial upgrading; while in the ecological dimension, the focus is on environmental protection and the harmonious coexistence of humanity and nature. Metaphors play a crucial role in shaping public perceptions of policy, reflecting specific values and socio-cultural contexts, facilitating cultural communication and understanding, and enhancing public responsibility and participation awareness.

## 1. Introduction

The historical background and evolution of the sustainable development concept reflect a profound reflection and proactive action by humanity regarding its developmental models. The defining concept of sustainable development was first systematically introduced in the 1987 Brundtland Report by the United Nations World Commission on Environment and Development (WCED). This was followed by the adoption of Agenda 21 at the 1992 United Nations Conference on Environment and Development and the introduction of the Millennium Development Goals (MDGs) in 2000, leading to the 2015 United Nations 2030 Agenda for Sustainable Development. The sustainable development philosophy has evolved from initial environmental protection to encompass integrated economic, social, and ecological development, addressing various areas such as natural environmental conservation, economic growth, protection of the planet, and the pursuit of peace and justice, and has become a global consensus and a guiding framework for action [1]. This concept encompasses three crucial dimensions: economic, ecological, and social, aiming to achieve a harmonious coexistence between human development activities and the natural environment, while ensuring the needs of both present and future generations are met [2–4].

The Olympic Games represent the most significant event in global sports. Over the centuries, they have transcended their origins as mere athletic competitions, evolving into a social, cultural, and economic phenomenon. Sustainable development has gradually become one of the principles of the Olympics. People view the organization of the Olympic Games from the perspective of sustainable development, and the Olympics have also emerged as a crucial platform for host nations to promote and practice the principles of sustainable development [5]. The Beijing Winter Olympics, as the first "dual Olympics city" in history, holds a unique international status and far-reaching influence [6]. Through official news coverage, the Beijing Winter Olympics has become an important window for China to display its sustainable development philosophy, conveying its efforts and commitments in economic transformation, ecological protection, and social harmony to the world [7]. The extensive use of metaphors in the reporting, as a powerful cognitive tool, has shaped the public’s cognitive framework for sustainable development [8, 9]. Metaphors not only enrich the expressive power of language but also concretize the abstract concept of sustainable development, playing a crucial role in conveying complex ideas and promoting the dissemination of values [10, 11]. However, there has yet to be a systematic study on how metaphors shape and communicate the economic, ecological, and social dimensions of sustainability in the news coverage of the Beijing Winter Olympics. An in-depth study of the metaphors in the news discourse of the Beijing Winter Olympics is of significant academic and practical importance for clarifying how major international events like the Olympic Games serve as vehicles for the dissemination of sustainable development concepts and analyzing how metaphors influence public sustainable development cognition.

This study, grounded in the theory of criticism metaphor analysis (CMA), extends metaphor analysis to the dimensions of society, economy, and ecology, thereby constructing a theoretical framework for the critical metaphor analysis of sustainable development. This framework will systematically analyze the metaphors in the news coverage of the Beijing Winter Olympics, revealing their mechanisms in mapping the concepts of sustainable development in society, economy, and ecology, as well as their multifaceted roles in conveying policy advocacy, ideology, cultural values, public participation and social motivation. Through this framework, the study aims to reveal the underlying mechanisms by which news discourse conveys the concept of sustainable development through metaphors, providing a new perspective for understanding the role of major international events in promoting the concept of sustainable development.

## 2. Literature review and theoretical foundation

### 2.1 The sustainable development in the Olympics

The Olympics, as the world’s largest sporting event, increasingly plays a significant role in promoting sustainable development. Over the past few decades, the organizers of the Olympics and the International Olympic Committee (IOC) have gradually integrated sustainability principles into all aspects of the event, including venue construction, event management, and post-event legacy utilization. Historically, the concept of "Green Olympics" was first introduced at the 1994 Lillehammer Winter Olympics, which emphasized reducing environmental pollution, conserving natural resources, and raising public awareness of environmental protection [12]. As the 21st century progressed, Olympic organizers paid more attention to sustainability; both the 2004 Athens Olympics and the 2008 Beijing Olympics developed comprehensive sustainability plans addressing all aspects from construction planning to event management [12]. With growing concern for environmental issues and social challenges, the modern Olympics have increasingly become an essential platform for practicing and promoting environmental, social, and economic sustainability.

Numerous scholars have researched how the Olympic Games promote environmental, social, and economic sustainability through their organization and implementation processes. Specifically, regarding social aspects, the hosting of the Olympics provides an opportunity to raise public awareness and participation in sustainable development. Yeerkenbieke [13] found that environmental education and community engagement activities during the Olympics can help enhance citizens’ environmental awareness and sense of social responsibility. Shi and Bairner [14] explored the impact of the Olympics on increasing sports participation in host countries, showing that with effective policy support and social mobilization, the Olympics can inspire more people to engage in sports activities, thereby promoting health and well-being in society. Economically, organizers of the Olympics are increasingly focusing on reducing the economic burden of the event through effective resource management and maximizing economic benefits. For instance, Yamamura [15] mentioned in her study of the Tokyo Olympics that despite cost overruns, optimizing resource allocation and improving operational efficiency can yield long-term economic benefits for host cities and countries. Karamichas [16] showed in his research on the economic impacts of the Olympics that they can also invigorate the local economy by promoting tourism and related industries. From an ecological perspective, the Olympics promote sustainable development practices by advocating for green technologies and environmentally friendly products. According to the study by He and Zhang [17], the green building standards and renewable energy projects implemented during the Olympics not only reduced the environmental impact of the event but also provided long-term environmental benefits to local communities. Tziralis [18] assessed the economic and environmental impacts of the 2004 Athens Olympics and pointed out that although the Athens Olympics promoted economic growth and employment in the short term, it also brought environmental pressures such as increased energy consumption and traffic pollution, underscoring the need for greater attention to environmental protection and resource management. Finally, Monica et al. [5] discussed the Winter Olympics from Vancouver 2010 to Sochi 2014, arguing that the Olympic Games played a positive role in promoting economic development, social inclusion and environmental protection of host cities and countries. In summary, the Olympics play an essential role in promoting environmental, social, and economic sustainability through their organizational and implementation processes. Despite the challenges, the Olympics continue to be a significant force in advancing global sustainable development goals.

### 2.2 Beijing Winter Olympics’ sustainable development practices

As the first Olympics hosted by a "double Olympic city" in history, the Beijing Winter Olympics not only holds historic significance in the realm of sports but also plays an essential role in promoting and practicing sustainable development. The Beijing Winter Olympics adhered to the principles of green, shared, open, and clean Olympic games, comprehensively advancing sustainable development practices through the adoption of green energy, promotion of ecological protection, encouragement of public participation, and enhancement of public awareness. Wang et al. [19] presented a case study on the sustainable land use and green ecology of the Beijing 2022 Winter Olympics venues. They assessed the spatial distribution and evaluated the legacy experience of the Olympic venues in terms of construction and sustainability. The study concluded that the Beijing Winter Olympic emphasized that the venues incorporated sustainable land management and green ecology concepts from the Olympic Movement, serving as a model for future development of Olympic infrastructure, natural sites, and resource management. Shi et al. [20] examined the spatial planning and land utilization of the Olympic venues, using the Beijing 2022 Winter Olympics venue legacy as a case study. They found that the Beijing 2022 Winter Olympics venues demonstrated a commitment to renewable energy supply and ecological protection, with minimal ecological damage and intensive reforestation activities. The study showed that the Beijing Winter Olympics contributed to a green urban economy development model driven by the Olympic Movement, highlighting the Games’ legacy in terms of societal value and resource scarcity. Zhang et al. [21] indicated that the hosting of the Winter Olympics brought economic growth and employment opportunities to the local area, while also invigorating the economy of host cities and regions by promoting tourism and related industries, resulting in long-term economic benefits. Li et al. [22] analyzed the impact of the Beijing Winter Olympics on China’s winter sports competition level, industry development, venue construction, and cultural dissemination. The study indicated that the Beijing Winter Olympics significantly promoted the popularization and development of winter sports in China, leading to an increase in public participation and the establishment of winter sports infrastructure.

The role of public participation and media communication in shaping the narrative and impact of the Beijing Winter Olympics is a significant aspect of sustainable development practices. Shi and Zhang [7] pointed out that during the preparation and hosting of the Beijing Winter Olympics, the media’s communication strategies and public acceptance worked in tandem. By emphasizing the themes of "Smart Olympics" and "Green Olympics," the event was positioned as a platform to showcase China’s technological prowess and green development concepts. Yeerkenbieke [13] conducted a survey to study the public’s views on the sustainability impacts of the Beijing Winter Olympics. The results showed that the public generally believed the Olympics had a positive impact on enhancing urban sustainability and improving residents’ quality of life, which included not only environmental improvements but also advancements in community development, cultural preservation, and social welfare. He et al. [23] conducted a study on public participation and information disclosure for environmental sustainability of the 2022 Winter Olympics through a questionnaire survey of residents in Beijing and Zhangjiakou. The findings indicated that while few respondents participated in the Olympics’ activities, there was a positive attitude towards the roles and functions of information and communication technologies (ICTs) in Beijing 2022. They considered that ICTs played a significant role in facilitating public awareness and collective action on environmental management and sustainability issues. Cui [24] noted that through the construction of news media narratives, the Beijing Winter Olympics showcased a national image of China characterized by peace, responsibility, environmental protection, openness, fairness, and cooperation. These narrative strategies not only enhanced public awareness of the Beijing Winter Olympics but also promoted support for China’s efforts in sustainable development.

In conclusion, the current research underscores the Beijing Winter Olympics’ significant role in advancing sustainable development and enhancing public awareness and participation in sustainable practices, setting a precedent for future Olympic events and urban development initiatives.

### 2.3 Metaphor in sustainable development discourse

Metaphor, as a complex linguistic phenomenon, transcends mere rhetorical devices by shaping cognitive processes through the mapping of familiar, concrete concepts (source domains) onto unfamiliar, abstract concepts (target domains) [25]. Metaphor is a multidimensional research object, with linguistic, cognitive, and communicative attributes, playing roles in rhetoric, conceptual understanding, and social communication [26, 27]. Metaphor is widely utilized in discourse such as news reporting, as they can concretize abstract concepts, making them more accessible and understandable to the public. For instance, the phrase "the road of social development" uses the concrete image of a road to symbolize the abstract concept of the process of social development. In the context of the Beijing Winter Olympics, metaphors have been strategically employed in news reporting to convey the complex narratives surrounding the event. Luo and Zhou [28] examined the metaphors in the opening ceremony of the Beijing Winter Olympics, revealing the presence of various conceptual metaphors, including themes of war, competition, and personification. These metaphors showcased China’s cultural confidence and articulated a hopeful vision for national reunification. Cai et al. [29] utilized a multimodal corpus to study the use of multimodal metaphors in the opening ceremony, finding that it constructed an image of China dedicated to cultural confidence, the building of a shared future for humanity, advanced scientific technology, and sustainable development. Bei [30] conducted a qualitative analysis of 1,673 news articles about the Beijing Winter Olympics, demonstrating that metaphorical language revealed media knowledge structures and emotional responses at a micro-level.

Metaphors play a multifaceted role in the discourse of sustainable development, serving as a bridge between abstract concepts and public understanding by endowing them with tangible imagery and emotional resonance [9, 31, 32]. Firstly, as a linguistic tool, metaphors in news reporting focus on how vivid imagery and symbolic language convey the complexities of sustainable development. For instance, comparing the Earth to a "sick patient" or a "weakened elder" can evoke public concern and empathy towards environmental issues. Research indicates that such metaphors can stimulate emotional resonance, thereby influencing readers’ attitudes and behaviors regarding environmental matters [10]. Moreover, metaphors play a significant role in shaping and disseminating values associated with sustainable development. By linking sustainability with positive concepts such as "health" and "harmony," metaphors in news reporting contribute to constructing a favorable societal vision and encourage public action towards achieving this vision [33]. Additionally, metaphors facilitate public understanding of practical aspects of sustainability, such as referring to green energy as "the energy of the future" or "clean energy." This not only communicates the necessity of energy transition but also underscores its urgency [11]. Allan [34] explored the risks and opportunities associated with the metaphor of "soil security," highlighting its significance in public understanding of reality and its broader connections with environmental, water, food, and energy security. Furthermore, Kopnina [8] examined the role of metaphors in environmental education and sustainability discourse, arguing that metaphors enable the transformation of unknown or incompletely understood emotions into coherent narratives, which are integral to social construction processes and emotional experiences. Mu et al. [35] employed multimodal metaphor theory to analyze ecological metaphors in the promotional video of the Beijing Winter Olympics, discovering that the video integrated the twenty-four solar terms of traditional Chinese culture, the spirit of the Olympics, and ecological elements to express positive natural and social ecological meanings, ultimately constructing a harmonious national ecological image.

Overall, existing literature highlights the critical role of metaphors in sustainability discourse, significantly influencing public perception and fostering environmental action. However, most studies have primarily focused on ecological metaphors, with few comprehensive investigations that encompass the social, economic, and ecological dimensions, especially regarding the Beijing Winter Olympics. Moreover, there is a lack of focus on news reporting within the discourse genre. Therefore, this paper aims to fill this gap by systematically exploring how metaphors in news reporting on the Beijing Winter Olympics promote the transmission of sustainable development concepts across social, economic, and ecological dimensions, thus providing a more comprehensive analysis of the Beijing Winter Olympics’ promotion of sustainable development from a metaphorical perspective.

### 2.4 Critical metaphor analysis theory

A systematic analysis of metaphors requires a supporting theoretical framework. Critical Metaphor Analysis (CMA) is a research method that combines cognitive linguistics with Critical Discourse Analysis (CDA), proposed by Charteris-Black in 2004 [36]. The core objective of CMA is to reveal how the use of metaphors in discourse reflects and shapes social ideologies, power relationships, and values. The development of this theory has enriched the field of metaphor studies and provided new perspectives for analyzing political, economic, and social discourse. The analytical process within the CMA framework typically involves three steps: metaphor identification, metaphor interpretation, and metaphor explanation. Metaphor identification refers to recognizing metaphorical expressions within specific texts; metaphor interpretation involves analyzing the relationships between the source domain and target domain that these metaphorical expressions rely on; and metaphor explanation further explores how these metaphors construct ideologies and values within particular socio-cultural contexts. These three steps align with the linguistic, cognitive, and social communicative attributes of metaphors.

Applying the CMA framework, several studies have shed light on the role of metaphors in shaping discourse across various domains, including economics, business, politics, and social media. Rojo López and Orts Llopis [37] apply CMA to a comparative study of metaphors in English and Spanish financial texts during the Global Systemic Crisis. Their analysis is based on financial articles from English journal *The Economist* and the Spanish newspaper *El Economista* published in 2007 and 2008. The study reveals how metaphors can frame economic realities differently based on socio-political factors. The authors demonstrate that metaphors serve as potent instruments for constructing narratives that align with specific political interests, thus shaping public perception of economic events. Koller [38] applies Critical Metaphor Analysis to examine metaphors in business magazine texts on mergers and acquisitions. Analyzing a 164,509-word corpus from four major business publications from 1997 to 2000, Koller finds a prevalent use of FIGHTING, MATING, and FEEDING metaphors, which together create a narrative of evolutionary struggle. This highlights the use of metaphorical models to reinforce ideological stances in media discourse. Lee [39] employs CMA to deconstruct 58 speeches by Singaporean government leaders on the importance of national education for national survival. The analysis uncovers eight metaphoric themes, with a focus on CONTAINER, BUILDING, REMEMBERING, and MORALITY. These themes, when situated within Singapore’s socio-political landscape, reflect the government’s efforts to maintain its hegemonic leadership through discourse. Lee’s research emphasizes the efficacy of CMA in uncovering the underlying propositions and agendas within societal discourse. Xu [40] conducts a comparative analysis of COVID-19 metaphors in Chinese and English social media using CMA, focusing on metaphors derived from Twitter and Weibo. While war and disaster metaphors are prevalent in both languages, English texts show a higher incidence of zombie metaphors, and Chinese texts feature more classroom metaphors. Xu attributes these differences to varying socio-historical factors and the active selection of metaphors by users to express their values and judgments. The study provides insights into how metaphors in social media reflect and influence cultural cognition during global crises.

The application of CMA in the analysis of sustainable development discourse primarily evaluates how metaphors construct and convey the concept of sustainability within discourse. Through CMA, researchers can unveil how metaphorical usage in sustainability discourse affects public perceptions and attitudes toward environmental issues, as well as how these metaphors serve specific political and economic agendas. Lin and Cao [41] applied CMA to analyze metaphor usage in the UN Protected Planet Report 2020 and the China Environment Yearbook, exploring the linguistic characteristics and cultural information conveyed through metaphor in Chinese and English ecological discourse. They found that metaphors related to war, journey, and living beings best reflect the differing ideologies within the ecological discourses of the two cultures. Skinnemoen [42] utilized CMA to examine metaphorical usage in climate change discourse, discovering frequent metaphors such as "climate change is movement," "environmentalism is war," and "environmentalism is a journey." These metaphors help simplify complex concepts, evoke emotional resonance, and potentially influence public understanding and action regarding climate change. Furthermore, by integrating ecological discourse analysis with CMA, researchers have developed a framework for ecological metaphor criticism that focuses on analyzing ecological value orientations and the macro-social context of discourse. Jin and Yu [43] conducted a comparative analysis of metaphors used in climate change speeches by the two major parties in the United States through the ecological metaphor criticism framework, and found that Trump tended to use metaphors related to business, war, personal property, and crime, reflecting a destructive ecological value orientation, whereas Biden employed metaphors more associated with threats, journeys, competition, and opportunities, indicating a more ambiguous ecological value perspective.

Although CMA has made significant progress in both the theoretical and practical aspects of discourse analysis related to sustainable development, existing studies still exhibit some shortcomings. Firstly, the theoretical frameworks tend to focus predominantly on ecological discourse analysis and lack a unified framework that integrates the social, economic, and ecological dimensions. This singular perspective may not fully capture the complexity and multidimensionality of metaphors associated with sustainable development. Additionally, metaphors of sustainable development often convey multiple layers of information concerning policies, ideologies, cultural values, and social behaviors, which have not been systematically discussed in existing research. Therefore, it is essential to construct a metaphor analysis framework that can integrate social, economic, and ecological dimensions, as it can provide a more comprehensive theoretical perspective to analyze and explain the role of metaphors in conveying sustainable development concepts.

**Fig 1:**
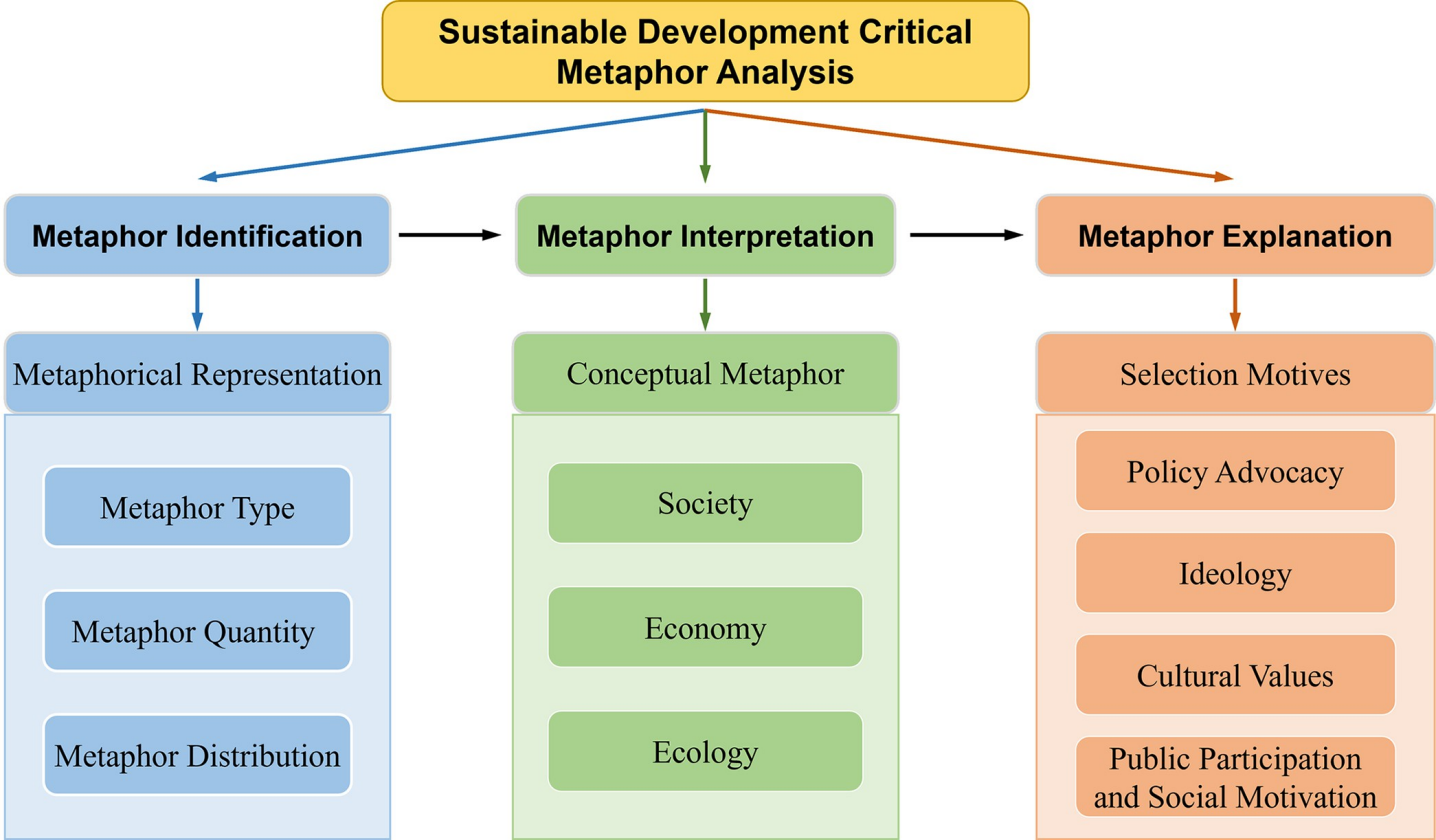
The critical metaphor analysis framework for sustainable development.

Consequently, this paper aims to explore the metaphors of sustainable development reflected in the news coverage of the Beijing Winter Olympics, using the social, economic, and ecological dimensions as the research lens. Given that the CMA has been applied in the analysis of discourses related to ecology, society, and economy, this study attempts to construct a critical metaphor analysis framework for sustainable development based on CMA. This framework facilitates a systematic analysis through three stages: identification, interpretation, and explanation of metaphors (see Fig 1). In the metaphor identification phase, high-frequency words related to metaphors of sustainable development in the news reports are identified through both quantitative and qualitative methods. The metaphor interpretation phase further analyzes how these metaphorical vocabularies form a cognitive framework for the public’s understanding of the Beijing Winter Olympics, centered around the three dimensions of society, economy, and ecology. The metaphor explanation phase provides an in-depth analysis of the social functions of metaphors from four dimensions: policy advocacy, ideology, cultural values, public participation and social motivation, revealing the central role of metaphors in constructing and disseminating the concept of sustainable development.

## 3. Materials and methods

### 3.1 Selection of news reports

The present study directs its scope towards the news coverage of the Winter Olympics by prominent Chinese news media outlets, namely "People’s Daily," "China Daily," and "Xinhua Net," which are recognized as influential party media outlets within China [44]. To encapsulate a holistic perspective of the Beijing Winter Olympics, a compilation of news reports was conducted, capturing the entire span of the Games from February 4th to February 20th, 2022. Employing "Beijing Winter Olympics/Winter Olympics/2022 Winter Olympics" as the key search term, the inquiry was executed within the official databases of the three newspapers, prioritizing articles that demonstrated close relevance to the Games in both form and substance. The articles were selected based on two principal criteria: firstly, the length of the report should be no less than 300 words, ensuring a comprehensive discussion; secondly, the content had to be markedly associated with the sustainable development, including society, economy, and ecology. The aggregate collection consisted of 127 news reports, accumulating to a total of 185,231 words, with a distribution of 57 from "China Daily," 47 reports from "People’s Daily," and 23 from "Xinhua Net," thereby constituting an extensive news discourse corpus centered on the Beijing Winter Olympics.

### 3.2 Identification and analysis of metaphors

The fusion of corpus linguistics with metaphorical theory has become a dominant approach in the field of metaphor analysis [45]. The Metaphor Identification Procedure (MIP) is employed for uncovering metaphorical usage within spoken or written text, discerning if a lexical item operates metaphorically within a specific context [46]. This research engaged MIP alongside AntConc 3.5.9 (2020 release) for the detection of metaphors [47]. The methodology encompassed: 1. A complete review of the news reports to grasp the overarching narrative; 2. The extraction of salient terms from the reports and their frequency analysis using AntConc; 3. An exhaustive examination of contextual meanings for these terms, compared with their basic meanings from the "Modern Chinese Dictionary," to identify metaphorical keywords; 4. A subsequent analysis of the metaphorical keywords’ frequency and their classification according to their source domains. The metaphor identification process was conducted independently by three researchers, including the author and two doctoral colleagues with expertise in Foreign Languages, all of whom received training in metaphor analysis. In the final phase of the metaphor analysis, they discussed any contentious terms and ultimately reached a consensus.

To systematically analyze metaphors within the news discourse, we employed the grounded theory method [48], which involves a structured coding process to categorize and summarize content into themes based on their universality. The coding process was meticulously executed in three distinct phases: (1) Open Coding: Initially, three researchers independently conducted open coding on the established corpus. This phase involved identifying and recording high-frequency metaphorical words and their corresponding sentences, categorizing them according to their target domains. (2) Axial Coding: Following open coding, axial coding was applied to the thematic high-frequency words. During this phase, we established generic relationships between these words based on the commonality of their target domains. Thirteen sub-themes emerged from this process, each representing a cluster of metaphors sharing similar target domain characteristics. This phase involved integrating and inducing metaphors into these sub-themes according to the principles of logical correlation, similar meaning, and repeated occurrence. (3) Selective Coding: The final stage entailed selective coding, wherein researchers further induced the 13 sub-themes into three overarching themes—society, economy, and ecology—based on their interrelatedness. The coding was initially conducted by three researchers in parallel, ensuring a thorough and independent analysis. Consistency across the classifications was sought through a meticulous cross-verification process among the researchers. A unified agreement on the categorizations was ultimately established through collaborative discussions at a roundtable.

## 4. Results and discussion

### 4.1 Metaphor identification results

**Fig 2:**
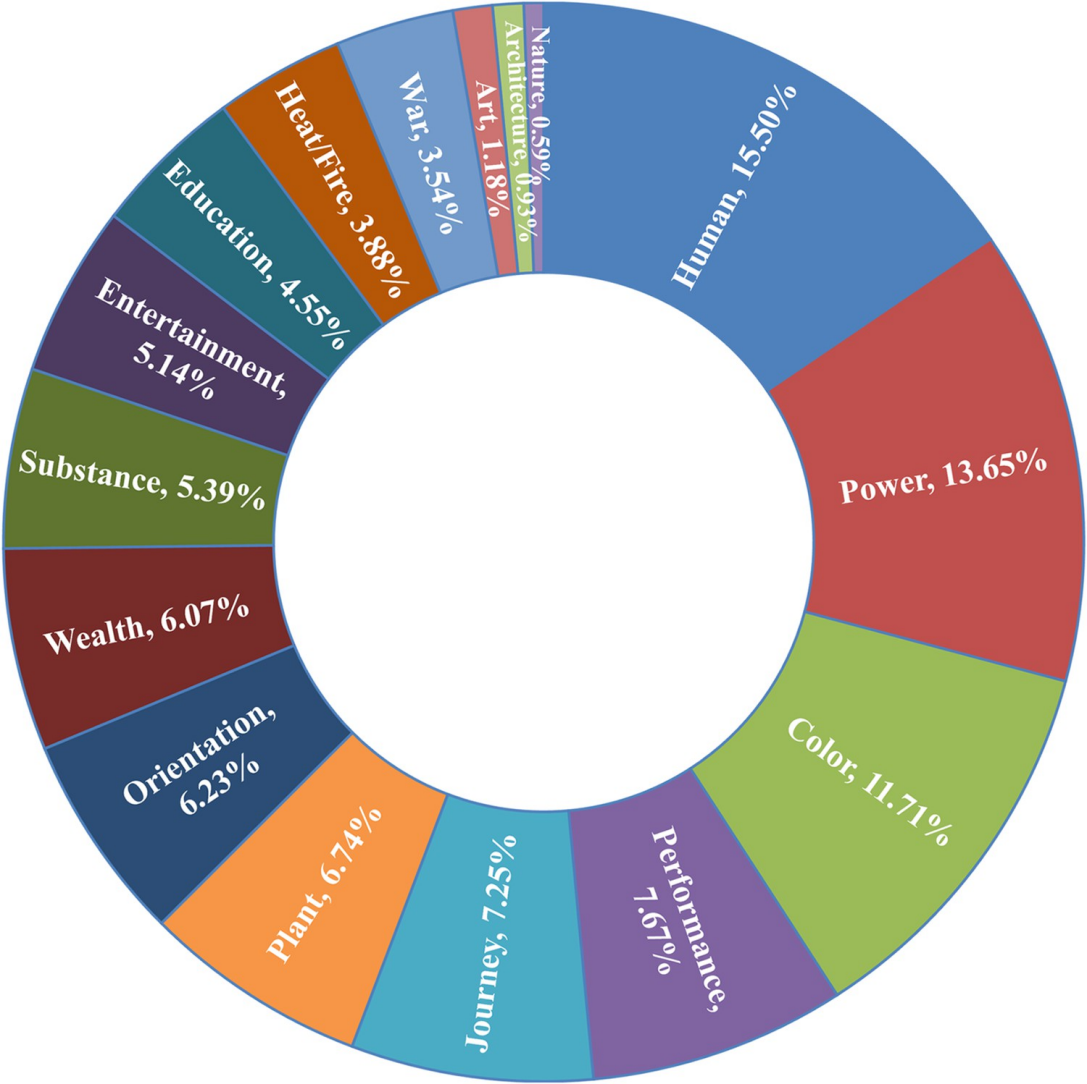
The types and quantity distribution of metaphor source domains in news discourse.

In the metaphor identification analysis of domestic mainstream news coverage of the Beijing Winter Olympics, a rich use of metaphorical language was discovered. According to the identification results, the frequency of metaphorical word usage in the news corpus reached 1,187 instances. These metaphorical expressions were categorized based on the source domain and quantitatively characterized in Fig 2. The results showed a diverse range of metaphorical language in news discourse, which could be grouped into 16 categories based on source domains. Among them, expressions using human (15.50%), power (13.65%), color (11.71%), performance (7.67%), and journey (7.25%) as metaphor source domains were relatively more frequent.

**Fig 3:**
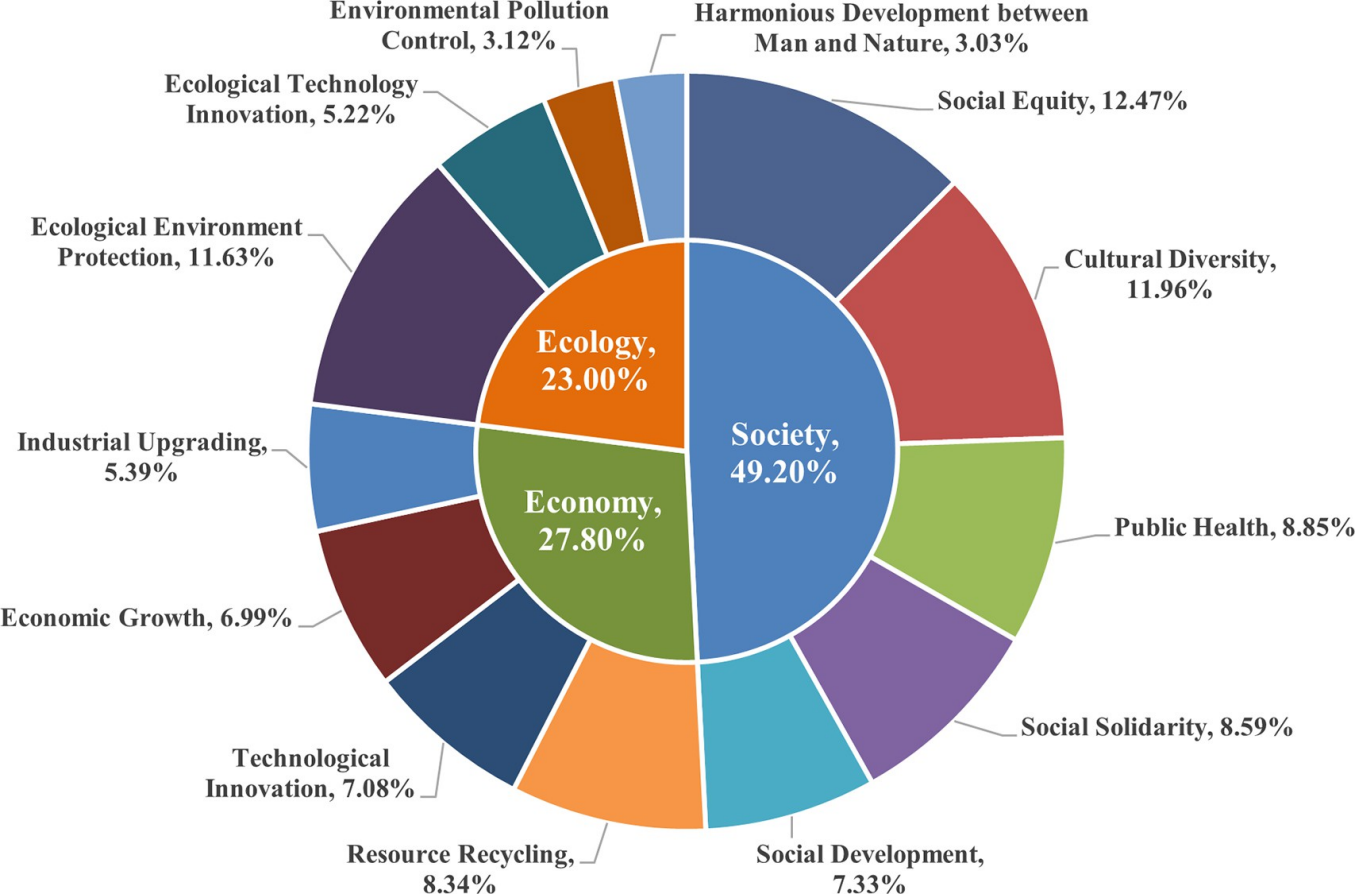
The types and quantity distribution of metaphor target domains in news discourse.

The quantitative characterization of metaphorical expressions in news discourse, categorized by target domain, is presented in Fig 3. It indicates that the target domain encompasses three topics: society (49.20%), economy (27.80%), and ecology (23.00%). Each theme includes 4–5 subtopics, which also serve as the direct target domains for the metaphors. These metaphors reflect how the news media shapes a specific perspective on the sustainable development concept of the Winter Olympics through metaphorical language. Next, an analysis will be conducted on the metaphorical words used in these subtopics and their sentences to provide a detailed interpretation of how metaphorical words map and shape the subthemes of the target domains.

### 4.2 Metaphor interpretation results

Metaphor interpretation will combine exemplar sentences to expound on the mapping relationships of conceptual metaphors under the three themes of society, economy, and ecology, as well as their sub-themes. Due to space constraints, the representative examples including their original Chinese versions and English translations placed in the supplementary material.

#### 4.2.1 Society

The news coverage of the Beijing Winter Olympics has intricately woven the concept of sustainable development within the societal context through the use of metaphors. As depicted in Table 1, the news discourse constructs the notion of sustainable society by leveraging metaphors that map across various source domains to target domains. The findings reveal that the target domains encompass social equity (25.34%), cultural diversity (24.32%), public health (17.98%), social solidarity (17.47%), and social development (14.90%). The metaphors, rich in frequency and diversity, illustrate the multifaceted dimensions and specific connotations of a sustainable society.

**Table 1:** Conceptual metaphors for sustainable society in news discourse.

Sub-topic/target domain	Source domain	High-frequency metaphoric words	Frequency
Social Equity (25.34%)	Power	带动(spur)	51
	Performance	舞台(stage), 角色(role)	44
	Plant	开放(openness)	39
	Light	阳光(sunshine)	9
	Journey	道(road)	5
Cultural Diversity (24.32%)	Entertainment	盛宴(feast), 故事(story)	58
	Education	书写(write), 篇章(chapter), 史册(annals of history)	54
	Performance	奏响(play), 乐章(symphony)	30
Public Health (17.98%)	Entity	闭环(closed-loop)	41
	War	战胜(overcome), 挑战(challenge), 抗击(fight against), 胜利(victory), 冲击(impact)	24
	Heat	温暖(warmth)	17
	Human	肆虐(raging)	12
	Nature	寒冬(harsh winter), 阴霾(gloom)	7
	Plant	蔓延(spread)	4
Social Solidarity (17.47%)	Human	共同体(community), 家庭(family), 携手(hand in hand)	90
	Journal	向前(moving forward)	7
	Performance	和音(harmony)	5
Social Development (14.90%)	Journey	前行之路(road forward), 脚步(step), 出发(setting off), 迈向(stepping towards)	48
	Light	照亮(illuminate), 光芒(light)	16
	Architecture	窗口(windows)	11
	Power	动能(impetus)	5
	Human	脉搏(pulse)	4
	Entity	加速器(accelerator)	3

*(1) Social equity*. The metaphorical language within the realm of social equity reflects the media’s portrayal of the Winter Olympics as a catalyst for inclusivity and equal opportunity. The phrase "achieving the goal of spurring 300 million people in winter sports" uses the metaphor "spurring" to illustrate how sports events can drive social participation and equal opportunity. In "The Winter Olympics in Beijing provided a fair stage for athletes from all over the world," the metaphor "stage" embodies the concept of fair competition, suggesting that athletes from diverse backgrounds can compete in an equitable environment. The metaphor of "sunshine" signifies the transparent and equitable competitive landscape, symbolizing a society free from shadows where everyone is treated equally. The expression "Fairness and justice pave the road for righteousness among people" maps the "road" to symbolize the path of fairness and justice, underscoring equity as a cornerstone of social progress. These metaphors not only emphasize the importance of equality but also highlight the role of the Winter Olympics in promoting social equity.

*(2) Cultural diversity*. Cultural diversity is vividly represented through metaphor, conveying the blending of various cultural expressions. The line "A series of Chinese elements narrate the uniquely crafted ’Oriental Stories‴ employs the storytelling metaphor to weave a rich cultural narrative. The phrases "playing a magnificent symphony of unity, peace, and friendship for all humanity" utilize the terms "playing" and "symphony" as metaphors to represent the harmonious integration of different cultures, akin to various notes collaborating to create a beautiful and harmonious cultural symphony—reflecting the diversity and inclusiveness of culture. The expression "The Beijing Winter Olympics is not only a grand sports event but also a cultural feast" invokes the idea of a "cultural feast" to portray the harmonious coexistence of diverse cultures during this global spectacle. The metaphor "written a new chapter in the history of international sports" suggests that the Olympics serve as a platform for cultural exchange and integration. These metaphorical frameworks emphasize culture as a shared banquet and narrative, enriching and diversifying the human experience.

*(3) Public health*. In the context of public health, metaphors in news coverage depict the secure measures of the Olympics as a bastion for safeguarding public health amidst the COVID-19 pandemic. The metaphor "raging" personifies COVID-19, while "spread" likens the virus to a burgeoning plant, portraying it as a formidable adversary and highlighting the seriousness of the health crisis. The "closed-loop" metaphor suggests a controlled and safe environment that protects participants from external threats, vividly illustrating how the Olympics ensure safety through stringent health management protocols. Use of war-related metaphors such as "overcome," "challenge," "fight against," "victory," and "impact" conveys a combative stance, framing public health efforts as a battle and indicating a proactive and resolute position against health threats, reinforcing the significance of public health in overcoming global challenges. The metaphor "harsh winter" signifies the difficulties brought upon humanity by the pandemic, underscoring its severe impact on daily life. Metaphor like "gloom" metaphorically represent the lingering effects of the pandemic, embodying the shadow and pessimism it casts, and suggesting the negative influence on global mental health and social atmosphere. Conversely, "warmth" symbolizes the comfort and hope the Olympics brings to those affected by the pandemic, representing a beacon of solace and encouragement, indicating that even during a public health crisis, the warmth of social unity and international collaboration can provide spiritual support. By integrating these metaphors into their reporting, the news media effectively narrates public health as a societal priority, illustrating a robust public health framework that serves as both a shield against adversity and a lighthouse of hope amidst challenges.

*(4) Social solidarity*. In the domain of social solidarity, metaphors serve as powerful tools for expressing unity and mutual support, which are crucial for social cohesion. The phrase "jointly build a harmonious and cooperative international family" uses the metaphor "family" to convey a shared identity and sense of belonging, emphasizing that the international community should unite to confront shared challenges. The metaphor "hand in hand" further emphasizes collective action and mutual assistance, conjuring an image of people moving forward together toward common goals. Metaphors such as "moving forward" and "harmony" underscore the importance of united progress, reflecting the central role of social solidarity in fostering collective advancement. The term "a community with a shared future for mankind" encapsulates the idea of social unity, highlighting the necessity of individual participation and contribution to create a harmonious society, illustrating the strength found in unity and cooperation. These metaphors stress the vital role of collective effort in building an interconnected and evolving society, with news media usage underscoring the significance of unity in achieving social objectives and enhancing social cohesion, fostering a sense of solidarity that transcends geographical and cultural boundaries.

*(5) Social development*. Social development is depicted through metaphors in reporting, illustrating the dynamic evolution and progression of society. "The road forward for humanity" symbolizes the continuous journey of societal development, reflecting the ongoing pursuit of growth and advancement, indicative of the essence of societal evolution. Metaphors such as "step" and "stepping towards" concretely portray the steady advancement of society on the path of sustained development. The "window" metaphor likens the Winter Olympics to a display showcasing China’s image, signifying that the Olympic Games serve as a portal for the world to observe and appreciate China’s societal development. The "accelerator" metaphor conveys the catalytic role of the Olympic venues in regional development, illustrating the Winter Olympics’ role in propelling societal progress. The "pulse" in "China’s robust development pulse" metaphorically represents the vitality of societal development, symbolizing the strong momentum and enduring energy of societal advancement. "light" and "illuminate" metaphors reflect the positive impact of the Winter Olympics on the path of societal development, symbolizing that the Games provide spiritual strength for societal progress, illuminating and guiding society towards greater openness, inclusiveness, and advancement. These metaphors vividly portray a society brimming with vitality, constantly moving forward, and heading towards a bright future. By linking societal development with the backdrop of the Winter Olympics, the metaphors convey the concept of societal development as a persistent pursuit, akin to the Olympic spirit’s relentless pursuit of excellence in the face of challenges.

#### 4.2.2 Economy

The news discourse constructs the concept of a sustainable development economy through various source domain metaphors, as shown in Table 2. These target domains include resource recycling (30.00%), technological innovation (25.45%), economic growth (25.15%), and industrial upgrading (19.39%). The use of metaphors not only highlights the diverse values of economic activities but also reflects a profound understanding of the principle of sustainable economic development.

**Table 2:** Conceptual metaphors for sustainable economy in news discourse.

Sub-topic/target domain	Source domain	High-frequency metaphoric words	Frequency
Resource Recycling (30.00%)	Wealth	遗产(legacy), 金山银山(mountains of gold and silver)	72
	Human	变身(shapeshift), 华丽转身(gorgeous turn)	19
	Power	动力(impetus)	5
	Entity	接力棒(baton)	3
Technological Innovation (25.45%)	Orientation	提升(raise), 高科技(high-technology)	44
	Human	智慧(smart), 智能(intelligent)	26
	Journey	迈向(step towards), 飞跃(leap forward), 路(road)	14
Economic Growth (25.15%)	Power	带动(drive), 动力(impetus), 推动(spur)	51
	Plant	开放(opening up), 成果(fruit), 绽放(bloom)	27
	Art	画卷(picture)	5
Industrial Upgrading (19.39%)	Power	带动(drive), 加速(accelerate), 驱动力(driving force), 推动(spur)	50
	Light	点亮(illuminate)	4
	Plant	成果(fruit)	4
	Journey	路(road)	3
	Entity	快车(express train)	3

*(1) Resource recycling*. Within the subtheme of resource recycling, metaphorical language deeply illustrates how the news media perceives the Winter Olympics as a paradigm for promoting resource circularity and the reuse of economic heritage. For instance, "Out of 13 venues in the Beijing area, 11 are considered ’legacy’ venues from the previous Summer Olympics," employs the metaphor of "legacy" to emphasize the reuse of stadiums, symbolizing the precious wealth that blends history with modernity. Metaphors like "shapeshift" and "gorgeous turn" vividly describe the reutilization and reconfiguration of Olympic venues, effectively conveying the concept of resource recycling and reuse. "The icy and snowy landscape is also a mountain of gold and silver," uses the metaphor of "mountains of gold and silver" to directly link natural landscapes with economic value, highlighting the potential for natural resources to be converted into economic assets. "The reuse of Olympic venues infuses these structures with new impetus," the metaphor of "infusing new impetus" shows the innovative use and ongoing contribution of Olympic legacies in the context of a new era. "Resource recycling is like the baton," the metaphor compares resource recycling to the passing of a baton, vividly expressing the legacy and ongoing commitment to environmental responsibility. These metaphors collectively paint a picture of sustainable development where resources are recycled and historical legacies are innovatively transformed, deeply reflecting the positive impact of the Winter Olympics in promoting resource recycling and the reuse of economic heritage.

*(2) Technological innovation*. In the subtheme of technological innovation, the use of metaphorical language highlights the Winter Olympics as a platform for showcasing China’s technological progress and advancements in intelligence. "The content of intelligent technology in international large-scale events has significantly raised in the past six months," here, "raise" is used metaphorically to signify the notable progress of technology on the international stage. Words like "smart" and "intelligent" metaphorically represent the advancement and innovation of technology, depicting the Beijing Winter Olympics as a platform that displays China’s technological prowess and spirit of innovation. "Stepping towards" metaphorically indicates China’s determined steps and continuous progress in economic development, technological strength, and international influence, conveying a positive image of a nation constantly surpassing itself and moving towards higher goals. "This is a technological leap forward," here, "leap forward" metaphorically signifies that the application of the technology has achieved a qualitative breakthrough, symbolizing the leapfrog development of technology and showcasing China’s strength and innovation in the application of high-tech. These metaphors emphasize the positive role of the Winter Olympics in promoting technological innovation, accelerating the application of technology, and facilitating international exchange, showcasing China’s rapid development in the field of science and technology and its profound insight into the future intelligent society.

*(3) Economic growth*. The economic growth subtheme vividly illustrates the driving effect of the Winter Olympics on economic development through a series of metaphorical expressions. "The coordinated development of the Beijing-Tianjin-Hebei region, drives regional poverty alleviation and revitalization," uses the metaphor of "drive" to symbolize an economic development model that radiates from a focal point to the entire area, emphasizing the Winter Olympics’ role in promoting economic integration in the region. "The deepening of reforms and opening up has borne much fruit," where "opening up" and "fruit" metaphorically represent the remarkable economic progress resulting from these policies. The metaphor of "bloom" likens China to a flower in full bloom on the world stage, showcasing the vitality and influence of China’s economic development and its growing attractiveness and competitiveness in the global economy. "A new picture" metaphorically suggests the fresh perspectives and boundless potential that the Winter Olympics bring to urban development, highlighting the positive impact of such international events on city infrastructure, image, and brand shaping. "illuminate" metaphorically portrays the Winter Olympics as a beacon, lighting up new pathways for integrated regional development and bringing new opportunities and momentum to the economic growth and collaborative advancement of the Beijing-Tianjin-Hebei area. These metaphors collectively outline a grand blueprint where the Winter Olympics stimulate economic growth, invigorate urban vitality, and enhance the national image, demonstrating the significant value of large-scale sports events in driving socio-economic progress.

*(4) Industrial upgrading*. In the subtheme of industrial upgrading, metaphors vividly depict the crucial role of the Winter Olympics in promoting regional economic development and industrial transformation. "The inauguration of the Jingzhang High-Speed Railway drives the construction of Zhangjiakou area," where "drive" metaphorically indicates the high-speed rail as an engine for economic development, propelling infrastructure construction and industrial growth along its route. "Driving force" metaphorically suggests that the Winter Olympics have accelerated collaborative regional development, fostering optimization of economic structures and industrial upgrading. "Explored a precise road for industrial development," where "road" metaphorically represents the new direction and possibilities for local economic development provided by the Winter Olympics, finding a development model that suits its own characteristics and achieving precise industrial positioning and optimization. "This is a vivid demonstration of the fruits in China’s industrial transformation and development in recent years," where "fruits" metaphorically reflect the Winter Olympics as a microcosm of China’s industrial transformation and innovation achievements, demonstrating significant progress in urban construction, technological innovation, and industrial development. "Express train" metaphorically illustrates the rapid development opportunities the Winter Olympics have brought to the ice and snow industry in Chongli, marking a splendid transition from an impoverished county to an international tourist destination. These metaphors collectively present the Winter Olympics as an important platform for promoting industrial development, accelerating regional economic integration, and driving social progress, highlighting its positive contribution to achieving sustainable economic growth.

#### 4.2.3 Ecology

News discourse constructs the concept of sustainable ecological development through vibrant metaphors, as shown in Table 3. These target domains cover ecological environment protection (50.55%), ecological technology innovation (22.71%), environmental pollution control (13.55%), and harmonious development between man and nature (13.19%). The use of metaphors not only highlights the multiple meanings of ecological protection but also deepens the understanding of the importance of ecological civilization construction.

**Table 3:** Conceptual metaphors for sustainable ecology in news discourse.

Sub-topic/target domain	Source domain	High-frequency metaphoric words	Frequency
Ecological Environment Protection (50.55%)	Color	绿色(green), 红线(red line)	94
	Orientation	低碳(low-carbon), 低能耗(low energy consumption)	30
	Human	共同体(community)	9
	Journal	旅(journey)	5
Ecological Technology Innovation (22.71%)	Color	绿色(green)	41
	Entity	覆盖(cover)	10
	Human	智能(intelligent)	5
	Entertainment	盛典(ceremony)	3
	Art	描绘(painting)	3
Environmental Pollution Control (13.55%)	War	保卫战(battle to defend), 兵力(forces), 战役(battle), 战争(war)	18
	Human	智慧(wisdom)	11
	Color	北京蓝(Beijing Blue)	4
	Journal	路(road)	4
Harmonious Development between Man and Nature (13.19%)	Performance	音符(musical note), 奏响(play), 乐章(symphony)	12
	Human	共同体(community)	8
	Plant	生根(taking root), 发芽(sprout)	6
	Art	笔触(brush strokes), 画卷(picture)	6
	Entity	增长点(growth point), 发力点(advantage point)	4

*(1) Ecological environment protection*. In the subtheme of ecological environment protection, the metaphorical language in news discourse profoundly reflects respect and maintenance for the natural environment, as well as the advocacy of green development concepts. The hosting of the Beijing Winter Olympics was not only a sporting event but also a concentrated display of China’s achievements in ecological civilization construction. The metaphor of "green" in "During the Olympic process in China, the characteristics of technology, intelligence, green, and frugality were highlighted" conveys the emphasis on environmental protection during the preparation of the Winter Olympics, embodying the concept of a green Olympics. "Low-carbon" and "low energy consumption" serve as directional metaphors, jointly constructing a clear direction pointing towards sustainable development and environmental protection. By concretizing and targeting environmental protection concepts, they express the Winter Olympics’ proactive stance in reducing environmental impact and promoting green development. "The preparation process of the Beijing Winter Olympics is like a low-carbon journey," where "journey" metaphorically compares the preparation to an ongoing process with the goal of achieving low carbon emissions and promoting clean energy, thereby reducing environmental impact. "Mountains, waters, forests, farmlands, and lakes form a community of life" deeply expresses the interdependence and close connection between various components of the natural ecosystem, emphasizing the integrity and systematic nature of ecological environment protection. "Ecological red lines, the baseline of ecology" use the color metaphor to vividly express the strict boundaries and principles that must not be violated in ecological environment protection, highlighting the importance of ecological security. Through these metaphors, the news media not only vividly conveys the efforts and achievements of the Beijing Winter Olympics in ecological environment protection but also promotes the concept of green development to the public.

*(2) Ecological technology innovation*. The subtheme of ecological technology innovation showcases how the Beijing Winter Olympics serves as a stage for promoting the development and application of environmentally friendly technologies. These metaphors not only highlight the key role of technological innovation in environmental protection but also reflect profound insights into future green technologies. Metaphors such as "green energy" and "green electricity" convey an environmentally friendly technological application concept, showing the environmental protection inclination in the technological and energy choices of the Winter Olympics. The metaphor of "intelligent snowmaking" indicates the tremendous potential of technological innovation in resource conservation and efficiency enhancement, representing the direct benefits brought by technological progress and mapping the trend of intelligent and precise ecological protection technology in the future. "The opening ceremony is a low-carbon and environmentally friendly green ceremony" metaphorically describes the opening ceremony as not only a cultural event but also a display of environmental protection actions, emphasizing the integration of environmental protection concepts in large-scale activities. "China’s innovative and active force in painting a green future," where "painting a green future" metaphorically refers to the environmentally friendly torch lighting method at the opening ceremony and symbolizes China’s determination and efforts in ecological technology innovation, as well as its commitment to sustainable development in the future. Through these metaphors, news discourse conveys the positive attempts and significant achievements of the Beijing Winter Olympics in ecological technology innovation and showcases China’s determination and actions in this field to the world.

*(3) Environmental pollution control*. In the subtheme of environmental pollution control, the metaphorical language in news discourse highlights a firm determination and action toward environmental governance and ecological balance. The metaphor "battle to defend the blue sky" likens environmental protection to a collective struggle requiring the participation and joint efforts of all citizens, highlighting the urgency and importance of pollution control and expressing a serious stance and proactive measures towards environmental pollution issues. War metaphors such as "forces," "battle," and "wars" emphasize the collective and strategic nature of pollution control, underscoring the importance of society-wide participation and pollution management at its source. The metaphor "Beijing Blue" vividly describes the clean air and blue skies achieved through pollution control measures, symbolizing the value placed on the outcomes of environmental management. "Ecological wisdom," as a metaphor, represents not only technological innovation but also a responsible attitude and culture towards the environment. The metaphor "road of greening" depicts a road of ecological protection and development that adheres to natural laws and the country’s actual conditions, emphasizing the coordinated unity of environmental protection and social development. Using these metaphors, news discourse profoundly communicates the consensus and efforts in controlling environmental pollution and the unwavering belief in environmental improvement through technological innovation and collective action, enhancing public awareness of environmental governance issues.

*(4) Harmonious development between man and nature*. In the subtheme of harmonious development between man and nature, metaphors deeply express the respect and protection of the natural environment by the Beijing Winter Olympics, as well as the concept of promoting ecological civilization through sports events. "The ecological practices of the Beijing Winter Olympics are like green musical notes, playing a symphony of harmonious coexistence between humans and nature." Here, the metaphors "green musical notes" and "symphony" compare the ecological protection actions of the Winter Olympics to a harmonious orchestra, emphasizing the importance of harmonious coexistence between humans and nature, and how each action is an essential note in this musical composition. The metaphor "community of shared destiny" highlights the inseparable connection between humanity and the natural world, reminding us of the need for collective action to protect our shared home in the face of global challenges. The metaphor "taking root and sprouting" in "the concept of green and low-carbon will take root and sprout in this land" not only indicates the long-term impact of the Beijing Winter Olympics on ecological protection but also foretells the continuous expansion and deepening of the green development concept in China and globally. "The Beijing Winter Olympics have painted vivid pictures with green brush strokes, showcasing the vision of harmonious coexistence between humans and nature." Here, "brush strokes" and "pictures" metaphorically liken the environmental actions of the Winter Olympics to artistic creations, expressing admiration for the beauty of nature and the pursuit of a harmonious vision. The metaphors "growth points" and "advantage points" emphasize that a sound ecological environment is not only the foundation for improving people’s quality of life but also an important reflection of the country’s image and soft power. Through these metaphors, news reports vividly convey the efforts and achievements of the Beijing Winter Olympics in promoting the harmonious development of man and nature, while also educating the public on the importance of ecological civilization construction and showcasing China’s proactive actions and beautiful vision in promoting harmonious development between man and nature to the world.

### 4.3 Metaphor explanation results

The metaphor explanation will reveal how metaphors profoundly influence and shape public understanding of sustainable development concepts. By analyzing how metaphors are strategically applied in policy advocacy, ideological construction, cultural value transmission, and stimulating public participation and social motivation, we uncover the key role of metaphors in shaping social cognition and advancing the sustainable development agenda.

#### 4.3.1 Policy advocacy

As an important channel for disseminating sustainable development policy advocacy, the news reports on the Beijing Winter Olympics profoundly influence public understanding and perception of policies through the selection and expression of metaphorical language [49]. In reporting on the Beijing Winter Olympics, the news media strategically employed a series of metaphors to effectively convey policy objectives and shape public cognition. In terms of conveying policy goals, metaphors such as "green technology" and "green energy" clearly communicate the specific objectives of the policies, namely achieving dual goals of environmental protection and economic development through technological and energy transformation. Additionally, describing the Winter Olympics as "a stage for fair competition among athletes from around the world" not only showcases the international and fair nature of the event but also conveys policymakers’ emphasis on equal opportunities. In shaping public cognition, the use of metaphors also plays a crucial role. For example, metaphors like "low carbon" and "low energy consumption" not only convey the efforts of the Winter Olympics in environmental protection but also stimulate public support for clean energy policies. Metaphors like "the battle to defend the blue sky" not only set public expectations for the effectiveness of environmental policies but also convey a firm determination to improve air quality. These metaphors concretize abstract environmental protection concepts, allowing the public to more intuitively perceive the positive changes brought about by the policies [32]. Through these metaphors, the news media and policymakers can ensure sustained public attention and understanding of policy objectives, creating a favorable public opinion environment for the smooth implementation of policies [50].

#### 4.3.2 Ideology

The news reports on the Beijing Winter Olympics contain specific ideologies regarding sustainable development, and the use of metaphors effectively disseminates these ideologies while shaping deep-seated values about sustainability in the public’s mind. The interaction between ideology and metaphor is a key mechanism for constructing and disseminating social values [51]. In the reporting of the Beijing Winter Olympics, metaphors such as "green development" not only convey environmental protection concepts but also embody a deeper ideology of pursuing a balance between economic and ecological interests. Such metaphors, through media dissemination, directly influence public understanding and attitudes toward sustainable development. For example, the war metaphor of "fighting against the COVID-19 pandemic" demonstrates the Chinese government’s determination to adhere to a "dynamic zeroing" policy, reflecting the values of "people first, life first." The use of metaphors shapes public cognition and collective consciousness, helping the news media to shape public values on sustainable development [52]. For instance, the sub-theme of harmonious development between man and nature reinforces public recognition and responsibility for ecological protection, resonating with the core values of ecologism. The use of these metaphors not only shapes public values but also strengthens the sense of responsibility for the global mission of sustainable development at a deeper level. Moreover, there is a close relationship between power discourse and metaphor construction. The use of metaphors plays an important role in reflecting and reinforcing social power structures, becoming a tool for different interest groups to express and pursue their goals [53]. For example, the metaphor of "green energy" may superficially focus on environmental protection, but at a deeper level, it may represent the interests and policy directions of specific energy industries, revealing the dominant ideology within the power structure.

#### 4.3.3 Cultural values

In the global event of the Beijing Winter Olympics, the inheritance and exchange of cultural values are particularly important. Metaphors embedded in cultural contexts are crucial for understanding and conveying cultural values [54]. For instance, metaphors such as “Oriental stories” and “cultural feast” not only reflect the uniqueness of traditional Chinese culture but also highlight the diversity and inclusiveness of Chinese culture through narrative and celebratory means. The use of these metaphors not only communicates the profound essence of Chinese culture to the world but also fosters mutual understanding and respect among different cultures. Additionally, the metaphor of "harmonious coexistence between humanity and nature" underscores the philosophical concept of the unity between heaven and humanity in traditional Chinese thought. This aligns with contemporary society’s emphasis on ecological civilization, reflecting a shared pursuit of environmental sustainability in cross-cultural communication. Moreover, the strategic use of metaphors effectively shapes and disseminates cultural values related to sustainable development [55]. For example, by depicting the Winter Olympics as a showcase of a "sunny, prosperous, open, and hopeful national image," the news media conveys not only China’s national identity but also an aspiration for health, vitality, and a positive future. Such metaphorical expressions facilitate the formation of social consensus and cultural identity. The metaphor of "playing a magnificent melody of unity, peace, and friendship for all humanity" not only emphasizes the Chinese cultural appreciation for harmony and diversity but also promotes dialogue and understanding among different cultures through its inclusive language. It underscores the importance of seeking consensus within diversity, deepening the recognition of the concept of harmonious development, and illustrating the positive role cultural values play in advancing societal progress.

#### 4.3.4 Public participation and social motivation

Public participation and social motivation are also important aspects that sustainable development metaphors aim to convey. The interplay between public feedback and metaphors contributes to the advancement of the sustainable development agenda. Social psychology indicates that an individual’s self-cognition and attitudes are the foundation of participatory behavior [56, 57]. For example, metaphors like "winning the battle to defend the blue sky" not only resonate with the public on environmental issues but also promote positive social feedback and participation. At the same time, the metaphor of "ecological wisdom" encourages public support for environmentally friendly technologies. In terms of group influence and social norms, the metaphor "humans and nature are a community of shared destiny" reinforces public group identity and promotes the formation of social norms that support sustainable development. Additionally, the metaphor "mountains, waters, forests, farmlands, and lakes form a community of life" not only strengthens the public’s understanding of the holistic and systematic nature of ecological protection but also fosters collective action. The news media constructs a positive feedback mechanism through these metaphors, motivating public participation in environmental protection and creating a self-reinforcing cycle, where positive public feedback further enriches and strengthens the expression and application of the metaphors. Moreover, social movements influence the choice and use of metaphors, which reflect collective aspirations for social change and sustainable development goals [58]. Metaphors such as "ecological red line" and "green development" are not only reflections of the current situation but also calls for social transformation, stimulating the public’s impetus to engage in sustainable development practices. The Olympic concept metaphor "green, shared, open, and clean" mirrors the societal demand for change, mobilizing public involvement in actions that promote sustainable development. Social movements, through metaphors like "the wind from Zhangjiakou lights up the lamps of Beijing," demonstrate the use of clean energy and technological innovation, stimulating public support and participation in technological advancements for sustainable development.

**Fig 4:**
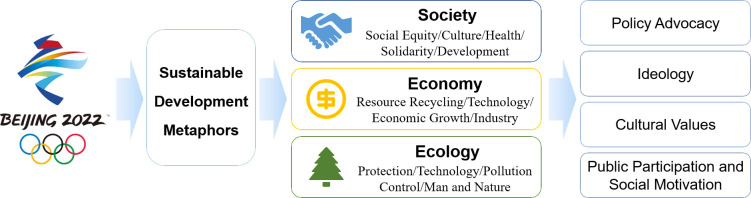
The role of sustainable development metaphors in the Beijing Winter Olympics.

Fig 4 illustrates the relationship between the Beijing Winter Olympics, sustainable development metaphors, and their role in shaping public perceptions. The Beijing Winter Olympics serves as a central platform, using metaphorical language in its news coverage to translate complex sustainability concepts into accessible narratives. These metaphors connect and activate three key dimensions of sustainability—social, economic, and ecological—each playing a crucial role in shaping public understanding of sustainable practices. In the social dimension, metaphors emphasize values such as fairness, cultural diversity, and community solidarity, which contribute to a sense of social cohesion. The economic dimension employs metaphors to highlight technological innovation, resource efficiency, and industrial upgrading, fostering sustainable growth and economic stability. Meanwhile, in the ecological dimension, metaphors focus on themes of environmental protection, ecological balance, and harmonious coexistence with nature, reinforcing public awareness of ecological preservation. These three dimensions work together to create a holistic approach to sustainable development, making the abstract goals of sustainability more relatable and inspiring public engagement, policy support, and cultural communication. This interconnected approach demonstrates how global events like the Beijing Winter Olympics can serve as powerful platforms for communicating sustainability, shaping public perceptions, and advancing a more sustainable future.

## 5. Conclusion

This study established a multidimensional critical metaphor analysis framework for sustainable development, systematically categorizing and interpreting the metaphors found in news coverage of the Beijing Winter Olympics. It deepens our understanding of how the news media employs metaphors to construct and disseminate sustainable development concepts. The research reveals that metaphors, as powerful linguistic tools, not only enrich the expression of news reports but also play a crucial role in fostering public understanding and acceptance of sustainable development ideas. From a social perspective, metaphors promote harmonious coexistence by emphasizing values such as fairness, diversity, and unity. In the economic dimension, metaphors highlight the recycling of resources, innovation-driven technology, and the upgrading and transformation of industries, reflecting the sustainability of economic development. Within the ecological dimension, metaphors focus on environmental protection and ecological balance, reinforcing the importance of ecological awareness and nature conservation. Additionally, metaphors serve important functions in shaping policy directions, conveying ideologies, communicating cultural values, and encouraging public participation, showcasing their multifaceted role in constructing social cognition.

The significance of this research lies in its integration of the social, economic, and ecological dimensions within a unified metaphor analysis framework for the first time, offering a new perspective on how major international events can act as vehicles for disseminating sustainable development concepts and providing new theoretical and methodological references for news discourse analysis. However, this study also has limitations; the exploration of the multidimensional social functions of metaphors is primarily based on corpus analysis and theoretical examination, lacking validation through empirical methods such as experiments or surveys. Future research could further investigate the role of metaphors in news coverage of large-scale sporting events through experimental or survey studies. Additionally, exploring how the use of metaphors in the context of new media can enhance the appeal and impact of sustainable development concepts will provide a richer theoretical and practical foundation for the development of sustainable communication strategies.

## Supporting information

S1 File (PDF)
