# Supplementary material for: Shaping sustainable perceptions: The role of metaphors in Olympic news discourse
Source: PLoS One. 2025 Jan 13;20(1):e0317380. doi: 10.1371/journal.pone.0317380 (PMC11729935; doi:10.1371/journal.pone.0317380)
Supplement: S1 File — (PDF) [file pone.0317380.s001.pdf]

Supporting information

Table S1 Examples of metaphorical sentences and English translations of the society topic

Sub-topic	Metaphorical Sentences	English Translations	Source
Social Equity	中国通过筹办冬奥会和推广冬奥运动，让冰雪运动进入寻常百姓家，实现了**带动** 3 亿人参与冰雪运动的目标。	Through organizing the Winter Olympics and promoting winter sports, China has brought snow and ice sports to the general public, achieving the goal of **spurring** 300 million people in winter sports.	Xinhua Net, 2022/2/5
	北京 2022 年冬奥会为全世界的运动员提供了一个公平竞技的**舞台**。	The 2022 Winter Olympics in Beijing provided a fair **stage** for athletes from all over the world.	China Daily, 2022/2/10
	作为万众瞩目的、担负着伟大**角色**的国际性体育盛会，奥林匹克运动会无疑成为了人类文化史上的一份杰作。	As an international sports event that garners global attention and bears a great **role**, the Olympic Games have undoubtedly become a masterpiece in the history of human culture.	China Daily, 2022/2/9
	北京冬奥会的成功举办，向世界展现了**阳光**、富强、**开放**、充满希望的国家形象。	The successful hosting of the Beijing Winter Olympics has showcased to the world a national image of **sunshine**, prosperity, **openness**, and hope.	People's Daily, 2022/2/17
	公平公正铺就人间正**道**，交流互鉴开创美好未来。	Fairness and justice pave the **road** for righteousness among people, and exchange and mutual learning create a bright future.	China Daily, 2022/2/9
Cultural Diversity	北京冬奥会不仅是一场体育盛会，也是一场文化**盛宴**。	The Beijing Winter Olympics is not only a grand sports event but also a cultural **feast**.	People's Daily, 2022/2/17
	“一系列中国元素讲述着匠心独运的‘东方**故事**’，呈现着中国文化和冰雪文化、奥运文化的完美融合”	A series of Chinese elements narrate the uniquely crafted 'Oriental **Stories**,' showcasing the perfect integration of Chinese culture with	People's Daily, 2022/2/17

		ice and snow culture and Olympic culture.	
	北京冬奥会**书写**了国际体育史上的新**篇章**。	The Beijing Winter Olympics has **written** a new **chapter** in the history of international sports.	People's Daily, 2022/2/12
	北京冬奥会一定会成为简约、安全、精彩的奥运会而载入**史册**。	The Beijing Winter Olympics will surely be recorded in the **annals of history** as a simple, safe, and splendid Olympic Games.	People's Daily, 2022/2/8
	中华文明与奥林匹克运动再度携手，**奏响**全人类团结、和平、友谊的华美**乐章**。	Chinese civilization and the Olympic Movement join hands once again, **playing** a magnificent **symphony** of unity, peace, and friendship for all humanity.	Xinhua Net, 2022/2/5
Public Health	北京冬奥会的**闭环**管理让各国各地区运动员感到安全、放心。	The **closed-loop** management of the Beijing Winter Olympics made athletes from various countries and regions feel safe and reassured.	People's Daily, 2022/2/20
	“中国为保障冬奥会顺利进行付出巨大努力，为运动员创造健康安全的比赛环境，也为我们共同**战胜**疫情传递出更多希望和信心。	China has made tremendous efforts to ensure the smooth progress of the Winter Olympics, creating a healthy and safe competitive environment for athletes, and conveying more hope and confidence in our joint **overcoming** of the pandemic.	People's Daily, 2022/2/15
	尼泊尔前驻华大使利拉·马尼·鲍德尔表示，在疫情跌宕反复的当下，北京冬奥会为人类战胜全球性**挑战**带来了希望。	Former Nepalese Ambassador to China, Leela Mani Paudyal, stated, "In the face of the fluctuating pandemic, the Beijing Winter Olympics has brought hope for humanity to overcome global **challenges**."	People's Daily, 2022/2/12
	中国人民以效率、决心和活力著称，无论是**抗击**新冠肺炎疫情还是举办北京冬奥会都已经证明了这点。	The Chinese people are known for their efficiency, determination, and vitality, which has been proven in both **fighting against** the COVID-19 pandemic and hosting the Beijing Winter Olympics.	People's Daily, 2022/2/6

	在史无前例的严峻疫情背景下取得的又一巨大**胜利。**	In the unprecedented severe pandemic context, the Beijing Winter Olympics is another great **victory**.	People's Daily, 2022/2/5
	在疫情**冲击**和日益复杂的世界格局下，人类分散则像雪花一样脆弱。	Amidst the pandemic's **impact** and the increasingly complex global landscape, humanity, when scattered, becomes as fragile as snowflakes in the face of adversity.	China Daily, 2022/2/18
	这场举世瞩目的体育盛会，不仅为身处疫情**寒冬**的各国人民带来了**温暖**与希望，更为动荡不安的世界注入了和平与团结的宝贵力量。	This globally anticipated sporting event has not only brought **warmth** and hope to people around the world enduring the **harsh winter** of the pandemic, but also infused the tumultuous world with the precious strength of peace and unity.	China Daily, 2022/2/19
	尽管新冠肺炎疫情仍在**肆虐**，但是冰雪运动带来的激情、欢乐和友谊仍然为全球人民共享。	Despite the **raging** COVID-19 pandemic, the passion, joy, and friendship engendered by winter sports continue to be a shared bounty among people across the globe.	People's Daily, 2022/2/15
	在新冠肺炎疫情**阴霾**仍未消退时举办北京 2022 年冬奥会，对于中国来说并非易事。	Organizing the Beijing 2022 Winter Olympics while the **gloom** of the COVID-19 pandemic still lingers is by no means a small feat for China	China Daily, 2022/2/15
	他们纷纷向本报记者表示，在疫情仍在全球**蔓延**的当下，北京冬奥会的举办振奋人心、意义重大。	They have expressed to our journalists that in the current climate where the pandemic continues to **spread** globally, the holding of the Beijing Winter Olympics is heartening and of significant import.	People's Daily, 2022/2/5
Social Solidarity	习近平主席以"诺亚方舟"为喻，传递中国推动构建人类命运**共同体**的决心。	President Xi Jinping uses the metaphor of "Noah's Ark" to convey China's determination to promote the construction of a **community** with a shared future for mankind.	Xinhua Net, 2022/2/7

	我们应该弘扬奥林匹克运动精神，团结应对国际社会共同挑战，践行真正的多边主义，共同建设和谐合作的国际大**家庭**。	We should promote the spirit of the Olympic Movement, unite to face the common challenges of the international community, practice true multilateralism, and jointly build a harmonious and cooperative international **family**.	Xinhua Net, 2022/2/5
	卡普拉洛斯指出，北京冬奥会将继续展示奥林匹克精神带领人们**携手**走出困境的决心。	Caprarossi pointed out that the Beijing Winter Olympics will continue to demonstrate the Olympic spirit's determination to lead people, **hand in hand**, out of adversity	People's Daily, 2022/2/7
	北京冬奥会开幕式体现了人性的光辉，也展现了各国人民团结**向前**的人类命运共同体理念和奥林匹克精神。	The opening ceremony of the Beijing Winter Olympics embodied the brilliance of humanity and also demonstrated the concept of a community with a shared future for mankind and the Olympic spirit, with people from all countries uniting and **moving forward.**	People's Daily, 2022/2/6
	在不同地方，通过不同方式，用不同语言，冬奥志愿者向各国各地区运动员和嘉宾展示友好，传递热情，唱响"一起向未来"的**和音**。	In different places, through different means, and in different languages, Winter Olympics volunteers show friendliness to athletes and guests from all countries and regions, convey enthusiasm, and sing the **harmony** of "together towards the future.	People's Daily, 2022/2/11
Social Development	生生不息的奥运之火，辉映"双奥之城"的光荣与梦想，**照耀**人类社会的**前行之路**。	The ever-burning Olympic flame reflects the glory and dreams of the "dual Olympics city," **illuminating** the **road forward** for humanity to overcome difficulties together and unite in cooperation.	Xinhua Net, 2022/2/4
	中国有越来越多孩子学习并参与冰雪运动，很像人类前进的**脚步**——跌倒了爬起来，再跌倒再爬起来，快乐地运动，再继续向前。	More and more children in China are learning and participating in ice and snow sports, much like the **steps** of human progress – falling and getting up, falling again and getting up again, exercising happily, and continuing to move forward.	People's Daily, 2022/2/6

	表达世界大爱与和平的音乐主题，展现北京冬奥会无穷力量和伟大精神，从冬奥**出发**，**迈向**世界。	The musical theme expressing universal love and peace demonstrates the endless strength and great spirit of the Beijing Winter Olympics, **setting off** from the Winter Olympics and **stepping towards** the world.	China Daily, 2022/2/9
	缓缓上升的"大雪花"，依然散发着耀眼的**光芒**，**照亮**每个人努力奋进、追逐梦想的人生之路，照亮全人类携手前行、共克时艰的美好明天。	The slowly rising "big snowflake" still emits a dazzling **light**, **illuminating** the path of life for everyone striving and pursuing dreams, and lighting up the beautiful tomorrow where all of humanity moves forward together to overcome difficulties.	Xinhua Net, 2022/2/4
	透过北京冬奥会这扇**窗口**，世人能看到 5000 多年文明的深厚积淀，也能一览当代中国日新月异的发展面貌。	Through the **window** of the Beijing Winter Olympics, the world can witness the profound accumulation of a civilization that spans over 5000 years, as well as the rapidly evolving face of contemporary China.	People's Daily, 2022/2/17
	无论参赛选手还是外国记者，不仅能感受到中国人民的热情好客、朝气蓬勃，更能直接触摸到中国强劲的发展**脉搏**，体会到中国智慧、中国方案为奥运、为世界注入的强劲**动能**。	Whether participants or foreign journalists, not only can they feel the warmth and vitality of the Chinese people, but they can also directly sense the strong **pulse** of China's development, and experience the robust **impetus** that Chinese wisdom and solutions bring to the Olympics and to the world	People's Daily, 2022/2/17
	北京冬奥会的每个赛区都像一个**加速器**，有力带动地区发展。	Each competition zone of the Beijing Winter Olympics is like an **accelerator**, effectively driving regional development.	People's Daily, 2022/2/14

Table S2 Examples of metaphorical sentences and English translations of the economy topic

**Sub-topic**	**Metaphorical Sentences**	**English Translations**	**Source**
Resource Recycling	北京赛区 13 个场馆有 11 个是当年夏季奥运会的‘**遗产**’”……比赛场馆反复利用、综合利用、持久利用，这些“中国经验”将是留给中国和世界的一份宝贵财富。	Out of 13 venues in the Beijing area, 11 are considered '**legacy**' venues from the previous Summer Olympics... The repeated, integrated, and sustainable use of these competition venues represents valuable 'Chinese experience' that will be a precious asset left for China and the world.	People’s Daily, 2022/2/4
	冰天雪地也是**金山银山**，乘上冬奥快车，崇礼的冰雪产业发展迎来重要机遇，从大山里的小镇，成为全球聚焦的滑雪胜地。	The icy and snowy landscape is also **a mountain of gold and silver**; boarding the express train of the Winter Olympics, Chongli's ice and snow industry has ushered in significant opportunities, transforming from a small mountain town to a globally focused skiing destination.	People’s Daily, 2022/2/14
	2008 年奥运会场馆经过改造再利用**变身**“双奥场馆”，这种场馆利用模式，汇集了往届奥运会在可持续方面的优点，为未来的奥运会提供了借鉴。	The venues from the 2008 Olympic Games, after renovation and reuse, have **shapeshifted** into 'dual Olympics venues'; this model of venue utilization has pooled the sustainable advantages of previous Olympic Games, providing a reference for future Olympic Games.	People’s Daily, 2022/2/10
	国家游泳中心完成了从“水立方”到“冰立方”的**华丽转身**。该场馆将在闭幕式后成为一个多用途的体育场馆。	The National Aquatics Center has completed a **gorgeous turn** from the 'Water Cube' to the 'Ice Cube'. This venue will become a multi-purpose sports facility after the closing ceremony.	China Daily, 2022/2/18
	奥运场馆的再利用，为这些建筑注入了新的**动力**，它们不仅是历史的见证，也是可持续发展理念的践行者。	The reuse of Olympic venues infuses these structures with new **impetus**; they stand not only as testaments to history but also as practitioners of the philosophy of sustainable development.	Xinhua Net, 2022/2/11

	资源循环利用，就像是冬奥赛场上的**<u>接力棒</u>**，一代代传递着环保的责任和使命。	The recycling of resources is akin to the **<u>baton</u>** in a winter Olympic relay race, passing on the responsibility and mission of environmental protection from generation to generation.	China Daily, 2022/2/12
Technological Innovation	从东京夏季奥运会到北京冬季奥运会，国际大型赛事中的智能技术含量在过去半年中的**<u>提升</u>**明显，中国的高新技术实力也在本届冬奥会上得到充分体现。	From the Tokyo Summer Olympics to the Beijing Winter Olympics, the content of intelligent technology in international large-scale events has significantly **<u>raised</u>** in the past six months, and China's high-tech strength has also been fully demonstrated at this Winter Olympics.	China Daily, 2022/2/4
	正因中国在广播和电信技术、工业方面如此成熟，我们才有机会使用这些**<u>高科技</u>**。	It is precisely because China is so mature in broadcasting, telecommunications technology, and industry that we have the opportunity to use this **<u>high-technology</u>**.	Xinhua Net, 2022/2/11
	北京冬奥会掀起冰雪运动的风尚潮流，中国高铁以**<u>智慧</u>**升级、**<u>智能</u>**创新，为蓬勃火热的冰雪运动再添场外支撑、踊跃动能，更好地展现了中国高铁新力量。	The Beijing Winter Olympics has set off a trend of ice and snow sports, and China's high-speed rail, with its **<u>smart</u>** upgrade and **<u>intelligent</u>** innovation, adds off-field support and dynamic momentum to the booming ice and snow sports, better showcasing the new strength of China's high-speed rail.	China Daily, 2022/2/9
	科技赋能生活、**<u>智能</u>**融入未来的理念有望更近人心，科技冬奥也将加速智能机器人的设计理念和技术细节的迭代更新。	The concept of technology empowering life and **<u>intelligent</u>** integration into the future is expected to win more hearts, and the technological Winter Olympics will also accelerate the iterative updates of the design concept and technical details of smart robots.	China Daily, 2022/2/14
	北京冬奥会不仅展现了中国的经济发展、科技实力和国际影响力，也标志着中国正在向体育强国不断**<u>迈进</u>**。	The Beijing Winter Olympics not only showcases China's economic development, technological strength, and international influence but	People’s Daily, 2022/2/5

		also signifies that China is continuously **stepping towards** becoming a powerful sports nation.	
	为了帮助运动员在北京冬奥会上取得最佳成绩，“冰丝带”首次采用了二氧化碳跨临界直冷制冰技术，这是一项技术上的**飞跃**。	To help athletes achieve their best results at the Beijing Winter Olympics, the "Ice Ribbon" has adopted carbon dioxide transcritical direct cooling ice-making technology for the first time, marking a technological **leap forward**.	China Daily，2022/2/17
	同我们国家的强国之路一样，中国冰雪运动也必须走科技创新之**路**。	Just like the path to becoming a powerful nation for our country, China's ice and snow sports must also follow the **road** of technological innovation.	People’s Daily, 2022/2/4
Economic Growth	京津冀协同发展，通过发展冰雪产业**带动**区域脱贫振兴，解决发展不平衡的问题。	The coordinated development of the Beijing-Tianjin-Hebei region, through the development of the ice and snow industry, **drives** regional poverty alleviation and revitalization, addressing the issue of unbalanced development.	People’s Daily, 2022/2/4
	北京冬奥会体现了区域一体化发展理念，是中国经济高质量发展的重要**动力**。	The Beijing Winter Olympics embodies the concept of regional integrated development and is an important **impetus** for high-quality economic development in China.	People’s Daily, 2022/2/14
	随着投资增加和基础设施的完善，张家口坚持生态优先，**推动**绿色发展，经济发展获得新动力，成为中国冬季运动产业的聚集地和旅游胜地。	With increased investment and improved infrastructure, Zhangjiakou adheres to ecological priority and **spurs** green development, gaining new momentum for economic growth and becoming a hub for China's winter sports industry and a popular tourist destination.	People’s Daily, 2022/2/14
	在两国元首引领下，哈中在政治、安全、经贸、人文等领域的合作取得丰硕**成果**，“光明之路”新经济政策	Under the guidance of the leaders of the two countries, Kazakhstan and China have borne rich **fruits** in cooperation across political, security, economic, and cultural fields. The alignment of	People’s Daily, 2022/2/9

	与共建“一带一路”倡议的对接开辟了广阔的合作前景。	Kazakhstan's "Bright Road" new economic policy with the joint "Belt and Road" initiative opens up broad prospects for cooperation.	
	“今天的中国跟 2008 年时的中国又不一样，深化改革**开放**让中国取得了巨大**成果**，实现脱贫攻坚胜利后的人民精神面貌焕然一新。	Today's China is not the same as it was in 2008; the deepening of reforms and **opening up** has borne much **fruit**, and the people's spirit has been revitalized after the triumph over poverty.	Xinhua Net, 2022/2/4
	中国在世界舞台上**绽放**精彩，成为世界经济发展的重要力量。	China has **bloomed** on the world stage and become an important force in world economic development.	People’s Daily, 2022/2/10
	北京冬奥会的举办，描绘了城市发展新**画卷**。	The hosting of the Beijing Winter Olympics has painted a new **picture** of urban development.	People’s Daily, 2022/2/14
Industrial Upgrading	京张高铁的开通进一步提升了北京和周边地区的联通性，也**带动**张家口地区建设世界级高水准的冰雪场馆及设施，大大**加速**北京周边地区的整体发展。	The inauguration of the Jingzhang High-Speed Railway has further enhanced the connectivity between Beijing and its surrounding areas, and **drove** the construction of world-class ice and snow venues and facilities in the Zhangjiakou region, significantly **accelerating** the overall development of the areas around Beijing.	People’s Daily, 2022/2/14
	北京冬奥会体现了区域一体化发展理念，是中国经济高质量发展的重要**驱动力**。	The Beijing Winter Olympics embodies the concept of regional integration and development, serving as a vital **driving force** the high-quality economic growth of China.	People’s Daily, 2022/2/14
	北京冬奥会的一项项建设成果，极大提升城市建设水平，**推动**文化、体育和旅游产业深度发展。	The construction achievements of the Beijing Winter Olympics have greatly enhanced the level of urban construction and **spurred** the in-depth development of culture, sports, and tourism industries.	People’s Daily, 2022/2/14
	北京冬奥会**点亮**京津冀区域一体化发展新机遇，**带动**基础设施、体育、休闲旅游等产业协同发展。	The Beijing Winter Olympics has **illuminated** new opportunities for the integrated development of the Jing-Jin-Ji region, **driving** the	People’s Daily, 2022/2/14

		coordinated development of industries such as infrastructure, sports, and leisure tourism.	
	借助北京冬奥会带来的机遇，张家口市张北县德胜村因地制宜，探索出一条精准的产业发展之**路**。	Leveraging the opportunities brought by the Beijing Winter Olympics, Desheng Village in Zhangbei County, Zhangjiakou City, has tailored its approach to local conditions and explored a precise **road** for industrial development.	People’s Daily, 2022/2/4
	首钢园区利用充满时代感的工业遗存，打造独具特色的冬奥赛场，同时初步形成科技产业集聚区，成为北京新的城市地标。“这是近年来中国产业转型发展**成果**的生动展示，体现了城市建设创新。	The Shougang Park, utilizing the industrial heritage imbued with a sense of the era, has created a unique Winter Olympic venue and has preliminarily formed a technology industry cluster area, becoming a new landmark of Beijing. This is a vivid demonstration of the **fruits** in China's industrial transformation and development in recent years, reflecting the innovation in urban construction.	People’s Daily, 2022/2/14
	乘上冬奥**快车**，崇礼的冰雪产业发展迎来重要机遇，从大山里的小镇，成为全球聚焦的滑雪胜地，并于2019 年退出贫困县序列。	Riding the **express train** of the Winter Olympics, Chongli's ice and snow industry has ushered in significant opportunities for development, transforming from a small town in the mountains to a globally focused skiing destination, and was removed from the poverty-stricken county list in 2019.	Xinhua Net, 2022/2/14

Table S3 Examples of metaphorical sentences and English translations of the ecology topic

Sub-topic	Metaphorical Sentences	English Translations	Source
	中国办奥过程中，突出科技、智慧、**<u>绿色</u>**、节俭特色，得到了世界各国的广泛认可。	Throughout the Olympic Games preparation in China, the emphasis on technology, intelligence, **<u>green</u>**, and frugality has garnered widespread recognition from countries around the world.	People’s Daily, 2022/2/7
	生态**<u>红线</u>**，生态底线。	Ecological **<u>red lines</u>**, the baseline of ecology.	People’s Daily, 2022/2/18
Ecological Environment Protection	北京冬奥会的竞赛场馆全部采用了绿色技术，100%使用绿色能源，体现出中国以冬奥为契机，积极引导人民群众开启绿色**<u>低碳</u>**生活模式，树立绿色**<u>低碳</u>**文明观，助力中国碳达峰碳中和宏伟目标的实现。	All the competition venues of the Beijing Winter Olympics have adopted green technologies and use 100% green energy, reflecting China's initiative to use the Winter Olympics as an opportunity to actively guide the public to start a green, **<u>low-carbon</u>** lifestyle, establish a green, **<u>low-carbon</u>** civilization concept, and support the achievement of China's ambitious goals of carbon peak and carbon neutrality.	China Daily，2022/2/17
	零碳排、**<u>低能耗</u>**、全热回收、无污染，冬奥会制冰造雪的新技术，代表着绿色、科技、环保的奥运理念，是北京冬奥会最美的底色。	Zero carbon emissions, **<u>low energy consumption</u>**, full heat recovery, and pollution-free, the new technology for ice and snow making at the Winter Olympics represents the green, technological, and environmentally friendly Olympic concept, and is the most beautiful background color of the Beijing Winter Olympics.	China Daily, 2022/2/18
	山水林田湖是一个生命**<u>共同体</u>**，人的命脉在田，田的命脉在水，水的命脉在山,山的命脉在土，土的命脉在树。	Mountains, waters, forests, farmlands, and lakes form a **<u>community</u>** of life; human life depends on the fields, fields depend on water,	People’s Daily, 2022/2/7

		water depends on mountains, mountains depend on soil, and soil depends on trees.	
	北京冬奥会的筹办过程如同一次低碳之**旅**，通过减少碳排放、推广清洁能源等方式，实现了环保与运动的完美融合。	The preparation for the Beijing Winter Olympics was akin to a low-carbon **journey**, achieving a perfect fusion of environmental protection and sports through reducing carbon emissions and promoting clean energy.	China Daily, 2022/2/8
Ecological Technology Innovation	北京冬奥会的竞赛场馆全部采用了绿色技术，100%使用**绿色**能源。	All the competition venues of the Beijing Winter Olympics utilized **green** technology and 100% green energy.	China Daily, 2022/2/17
	奥运史上首次实现全部场馆**绿色**电力全**覆盖**，被形象地称为"张家口的风点亮了北京的灯。	For the first time in Olympic history, all venues were **covered** by **green** electricity, vividly described as "the wind of Zhangjiakou lighting up the lamps of Beijing."	People's Daily, 2022/2/4
	冬奥会雪上场地采用**智能**造雪，相对于传统造雪，可节约水资源消耗约 20%。	The Winter Olympic snow venues used **intelligent** snow-making, which can save about 20% of water resource consumption compared to traditional methods.	People's Daily, 2022/2/11
	开幕式是低碳环保的**绿色盛典**。备受关注的点火仪式，也正因这一理念显得有些与众不同。	The opening ceremony was a low-carbon and environmentally friendly **green ceremony**. The much-anticipated lighting of the torch ceremony was also distinctive due to this philosophy.	China Daily, 2022/2/5
	北京冬奥会开幕式上，火炬点燃方式独特新颖，让我们看到中国用绿色**描绘**未来的创新力和行动力。	At the opening ceremony of the Beijing Winter Olympics, the unique and innovative way the torch was lit showed China's innovative and active force in **painting** a green future.	People's Daily, 2022/2/11

Environmental Pollution Control	坚持全民共治、源头防治，持续实施大气污染防治行动，打赢蓝天**保卫战**。	Adhering to public governance and source control, we continue to implement actions to prevent and control air pollution and win the **battle to defend** the blue sky.	People's Daily, 2022/2/16
	要集中优势**兵力**，动员各方力量，群策群力，群防群治，一个**战役**一个战役打，打一场污染防治攻坚的人民**战争**。	We must concentrate our superior **forces**, mobilize all parties, brainstorm collectively, and work together to prevent and control pollution, fighting a people's **war** against pollution that focuses on one **battle** at a time.	Xinhua Net, 2022/2/6
	北京冬奥会的生态**智慧**在多个方面得到应用，从节能技术到资源循环利用，都体现了对环境的深切关怀。	The ecological **wisdom** of the Beijing Winter Olympics has been applied in many aspects, from energy-saving technology to resource recycling, reflecting deep concern for the environment.	Xinhua Net, 2022/2/8
	整个京津冀及周边地区也开展协同共治、联防联控……下功夫、花心思，带来的不仅是 2021 年北京空气质量创历史最优、首次全面达标，还有冬奥期间举世瞩目的"**北京蓝**"。	The entire Beijing-Tianjin-Hebei region and its surrounding areas have also carried out collaborative governance and joint prevention and control...with great effort and thoughtfulness, what has been achieved is not only the best historical air quality in Beijing in 2021, the first time to fully meet the standards, but also the world-attention "**Beijing Blue**" during the Winter Olympics.	People's Daily, 2022/2/12
	一条符合自然规律、符合国情地情的绿化之**路**。	A **road** of greening that complies with natural laws and the national and local conditions.	People's Daily, 2022/2/16
	北京冬奥会的生态实践如同绿色**音符**，**奏响**了人与自然和谐共生的**乐章**。	The ecological practices of the Beijing Winter Olympics are like green **musical notes**, **playing** a **symphony** of harmonious coexistence between humans and nature.	Xinhua Net, 2022/2/13

Harmonious Development between Man and Nature	新冠肺炎疫情告诉我们，人与自然是命运**共同体**。	The COVID-19 pandemic has taught us that humans and nature are a **community** of shared destiny.	China Daily, 2022/2/14
	北京冬奥会的绿色理念与实践，不仅为冬奥会举办树立起新的标杆，更是为可持续发展和生态环境保护留下绿色遗产，绿色低碳理念会在这片土地上**生根发芽**。	The green philosophy and practices of the Beijing Winter Olympics have not only set a new benchmark for hosting the Winter Olympics but also left a green legacy for sustainable development and ecological and environmental protection; the concept of green, low-carbon will **take root and sprout** on this land.	China Daily, 2022/2/17
	“绿色、共享、开放、廉洁”的办奥理念落地**生根**，两地三赛区交出冬奥会筹办和本地发展两份优异答卷。	The Olympic concept of "green, shared, open, and clean" has **taken root**, with three competition zones within two regions delivering two excellent reports to both the preparation of the Winter Olympics and local development.	Xinhua Net, 2022/2/4
	在生态的画布上，北京冬奥会以**绿色笔触**绘制出一幅幅生动的**画卷**，展现了人与自然和谐共处的愿景。	On the canvas of ecology, the Beijing Winter Olympics has painted vivid **pictures** with **green brush strokes**, showing the vision of harmonious coexistence between humans and nature.	China Daily, 2022/2/15
	让良好生态环境成为人民生活的**增长点**、成为展现我国良好形象的**发力点**。	Let a good ecological environment become a **growth point** for people's lives and an **advantage point** for showing our country's good image.	Xinhua Net, 2022/2/8
